# Contribution of Priority PAHs and POPs to Ah Receptor-Mediated Activities in Sediment Samples from the River Elbe Estuary, Germany

**DOI:** 10.1371/journal.pone.0075596

**Published:** 2013-10-11

**Authors:** Jens C. Otte, Steffen Keiter, Christopher Faßbender, Eric B. Higley, Paula Suares Rocha, Markus Brinkmann, Dierk-Steffen Wahrendorf, Werner Manz, Markus A. Wetzel, Thomas Braunbeck, John P. Giesy, Markus Hecker, Henner Hollert

**Affiliations:** 1 KIT – Karlsruhe Institute of Technology, Institute of Toxicology and Genetics, Karlsruhe, Germany; 2 University of Heidelberg, Centre of Organismal Studies, Heidelberg, Germany; 3 RWTH Aachen University, Institute for Environmental Research, Aachen, Germany; 4 Department Bio-Chemistry and Ecotoxicology, Federal Institute of Hydrology, Koblenz, Germany; 5 Department of Animal Ecology, Federal Institute of Hydrology, Koblenz, Germany; 6 University of Koblenz-Landau, Institut für Integrierte Naturwissenschaften, Koblenz, Germany; 7 Toxicology Centre, University of Saskatchewan, Saskatoon, Canada; 8 School of the Environment & Sustainability and Toxicology Centre, University of Saskatchewan, Saskatoon, Canada; 9 Department of Biomedical Veterinary Sciences and Toxicology Centre, University of Saskatchewan, Saskatoon, Saskatchewan, Canada; 10 Department of Zoology and Center for Integrative Toxicology, Michigan State University, East Lansing, Michigan, United States of America; 11 Department of Biology and Chemistry, and State Key Laboratory in Marine Pollution, City University of Hong Kong, Kowloon, Hong Kong, SAR, China; 12 School of Biological Sciences, University of Hong Kong, Hong Kong, SAR, China; East Carolina University, United States of America

## Abstract

The estuary of the River Elbe between Hamburg and the North Sea (Germany) is a sink for contaminated sediment and suspended particulate matter (SPM). One major concern is the effect of human activities on the hydrodynamics, particularly the intensive dredging activities in this area that may result in remobilization of sediment-bound pollutants. The aim of this study was to identify pollutants contributing to the toxicological risk associated with re-suspension of sediments in the Elbe Estuary by use of an effect-directed analysis that combines chemical and biological analyses in with specific fractionation techniques.

Sediments were collected from sites along the Elbe Estuary and a site from a small harbor basin of the Elbe Estuary that is known to be polluted. The sixteen priority EPA-PAHs were quantified in organic extracts of sediments. In addition, dioxin equivalents of sediments were investigated by use of the 7-ethoxyresorufin *O*-deethylase assay with RTL-W1 cells and the Ah receptor-mediated luciferase transactivation assay with H4IIE-*luc* cells.

Quantification of the 16 priority PAHs revealed that sediments were moderately contaminated at all of the sites in the Elbe River Estuary (<0.02–0.906 µg/g dw). Sediments contained relatively small concentrations of dioxin equivalents (Bio-TEQ) with concentrations ranging from 15.5 to 322 pg/g dw, which were significantly correlated with dioxin equivalents calculated based on toxicity reference values and concentrations of PAH. The concentration of Bio-TEQ at the reference site exceeded 200,000 pg/g dw. In a potency balance the 16 PAHs explained between 47 and 118% of the Bio-TEQ in the luciferase assay, which can be explained by the constant input of PAHs bound to SPM from the upper course of the Elbe River into its estuary. Successful identification of a significant portion of dioxin-like activity to priority PAHs in complex environmental samples such as sediments has rarely been reported.

## Introduction

Sediments and suspended particulate matter (SPM) are often contaminated with complex mixtures of toxicants and represent sinks and potential sources for lipophilic pollutants [1]. Pollutants of concern in sediments include moderately to strongly lipophilic chemicals such as polycyclic aromatic hydrocarbons (PAHs), polychlorinated biphenyls (PCBs), polychlorinated dibenzo-*p*-dioxins (PCDDs), and polychlorinated dibenzofurans (PCDFs) and polychlorinated napthalenes (PCNs) [2], [3]. Some of these toxicants have been previously shown to be potentially hazardous to wildlife and humans. PCDDs, PCDFs, PCBs and other halogenated aromatic hydrocarbons (HAHs) and PAHs can be toxic to fish, especially to early life stages, and some are known to act through aryl hydrocarbon receptor (AhR)-mediated pathways [4], [5], [6]. In the environment, dioxin-like compounds rarely occur alone, but are typically present in mixtures of PCDDs, PCDFs, PCBs [7], PCNs [8] and other HAHs [9] with a wide spectrum of AhR-binding affinities. HAHs have been demonstrated to induce a number of toxic responses in vertebrates, including hepatotoxicity, body weight loss, thymus atrophy, impairment of immune responses, reproductive toxicity and modified thyroid metabolism, teratogenicity and carcinogenesis [10].

The estuary of the Elbe River between Hamburg and the North Sea (Germany) is a deposition zone for contaminated SPM originating in the upper course of the river. Due to alteration of the hydrodynamics of the Elbe River estuary, large amounts of SPM from the upper course of the river and marine SPM from the North Sea mix and settle in the estuary. As a consequence, dredging is required to keep the port of Hamburg accessible and open for marine vessels. Contamination of potentially polluted dredged material has to be assessed, and appropriate dumping sites identified [11]. The input of polluted SPM into the estuary is continuously monitored at permanent sampling points “Bunthaus” (river kilometer 610) and “Seemannshöft” (river kilometer 629). Since the early 1990s SPM collected at these locations revealed constant small but significant concentrations of selected priority contaminants such as HCB, copper (Cu), cadmium (Cd), mercury (Hg), lead (Pb), and zinc (Zn) bound to SPM from the upper course of the River Elbe [12] (all level of sediment contamination are according to [13]). In contrast, loads of PAHs, PCDD/Fs, and PCBs bound to SPM originating from the upper course of the river have decreased significantly since the early 1990s, but have remained constant at current concentrations since the mid ‘90s [12] or in some cases have even increased slightly since then [11], [14]. Direct emission of organic pollutants to the estuary by point sources other than SPM from the upper course can be mostly excluded except for minor emissions by old and characterised contamination sites in the vicinity of the port of Hamburg. In total, emission by the Hamburg area to the estuary corresponds approximately to less than 5% of the total annual load of particle-bound pollutants measured at Schnackenburg (river kilometer 475) for the upper course's input into the lower Elbe river [11]. Therefore, the source of PAHs, PCDD/Fs, and PCBs pollution in the Elbe river estuary is due to the input of contaminated SPM from the upper course of the river [12]. Elbe Estuary is dominated by transport of sediments and SPM between the North Sea and the River itself. This process is dominated by upstream transport of sediments of all fractions along the shallow water and especially of small-gained material from the North Sea to farther upstream of the port of Hamburg. SPM is transported upstream during phases with less low run-off, such as during spring tide. Additionally, salinity and along with this other physicochemical parameters of the sediments in the estuary varies depending on: 1) absolute location between the North Sea and the limnic parts of the Elbe River or the estuary and 2) seasonally changing run off from the limnic upper course of the river [11]. In total, Elbe Estuary is a dynamic system which differs from the limnic part of the Elbe River.

In fall 2006, the Elbe estuary (Germany) was subjected to an interdisciplinary study to identify the potential burden of sediments for designation of possible dumping sites for dredged material [11]. A total of 48 sampling sites were investigated from the limnic zone at river-km 634 near the port of Hamburg to the brackish-marine milieu at river-km 730 at Cuxhaven, where the Elbe River opens into the North Sea. Sediments were not taken from dredging or dumping sites. Besides hydro-morphological and chemical parameters of the sediments, abundances of zoobenthic and macrozoobenthic organisms were examined. To determine the ecotoxicological hazard potential of sediments and possible negative effects on local species, several standardized bioassays with marine and freshwater species were conducted on sediment pore waters and elutriates: Luminescent bacteria test with *Vibrio fischerii* (DIN EN ISO 11348-3), freshwater algae test with *Desmodesmus subspicatus* (DIN 38412-33), acute *Daphnia* toxicity test (DIN 38412-30), marine algae test with *Phaedactylum tricomutum* (DIN EN ISO 10253), and amphipod toxicity test with *Corophium volutator* (DIN EN ISO 16712). Surprisingly, these standardized assays at the organism level revealed apparent toxicity at some sites [15]. In contrast, population of fishes have been reported to be decreasing in the river Elbe estuary since the early 20th century, and were in part attributed to exposure to chemical pollutants [16]. Effects frequently observed in individuals collected from this reach of the river since the late 1980s and early 1990s exhibited cell damage and tumors as well as incidences of embryological malformation that are indicative of exposures with contaminants such as dioxin-like chemicals and genotoxic PAHs [17].

Given the constant input of SPM-bound PAHs and HAHs to the estuary, the need for risk assessment of sediment dredging activities in the Elbe estuary, and the finding that classical bioassays at the organismic level indicated toxicity in at least some locations, more susceptible sub-organismic assays based on mechanism-specific endpoints were applied. These might then serve as biomarkers of PAH and contamination with other dioxin-like HAHs; however, these sub-organismic biotests should closely mimic the *in vivo* response of an organisms of interest [9]. Therefore, the objective of this study was to estimate the hazard posed by AhR-agonists bound to sediments at selected sites along the river Elbe estuary. Based on chemical, hydro-morphological and ecotoxicological data collected during previous studies [11], sediments between river kilometers 634.0 and 680.0 were taken for investigation at the sub-organismal level, and biotest data were related to chemical data and standard toxicity tests. Selected sediment samples were tested for the induction of dioxin-like potency in two different *in vitro* cell assays representative of different classes of vertebrates [18]: The H4IIE-*luc* rat hepatoma cells which form a transactivation assay [19] and the fish cell line (RTL-W1 rainbow trout liver fibroblasts [20] were chosen to measure the relative potency of sediment extracts expressed as 2,3,7,8-tetrachlorodibenzo-*p*-dioxin (TCDD) equivalent concentrations (Bio-TEQs). These two assays were selected to characterize AhR-pathway activation (H4IIE.*luc*) and Cyp1A1 protein activity (EROD-activity; RTL-W1) in mammalian and fish cells, respectively.

To further characterize the origin of AhR- agonistic potency observed in the *in vitro* cell systems, multilayer fractionation of organic extracts of sediments was used to remove acid-degradable compounds, such as. To determine the proportion of the Bio-TEQs contributed by PAHs as well as more refractory compounds such as PCDDs/PCDFs, PCBs and PCNs, a potency balance was conducted [21]. Because most of the potency of AhR agonists could be attributed to acid-degradable compounds, it was likely that the Bio-TEQ were most likely due to the presence of PAHs so concentrations of selected PAHs were determined. To this end, concentrations of 2,3,7,8-TCDD equivalents measured by use of the bioassay (Bio-TEQ) were compared with the concentrations of 2,3,7,8-TCDD equivalents predicted by Chem-TEQs calculated as the sum of the product of concentrations of PAHs multiplied by their respective bioassay-specific relative potency factors (ReP) [22], [23], [24]. Comparison of Bio-TEQ and Chem-TEQ not only allows for the identification of substance classes and their possible contribution to the biological effect but also allows to compensate for uncertainties of both techniques simply by calculating the percentage of unknown non-priority pollutants in the sediments [25], [26].

## Materials and Methods

### 2.1 Sediment samples

**Figure 1:**
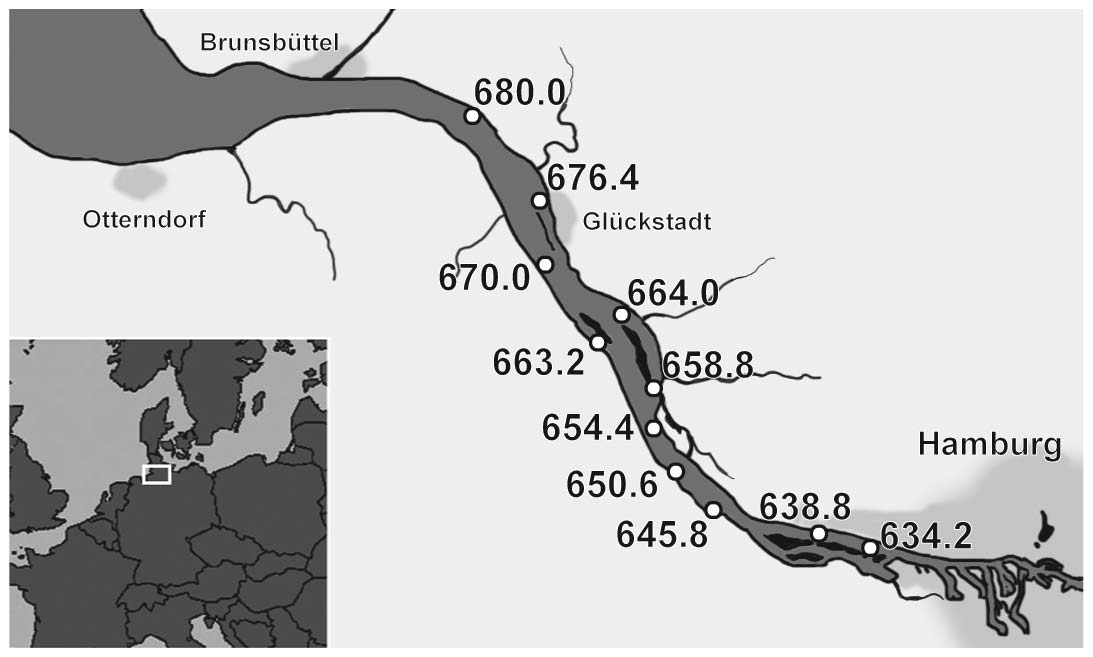
Sampling sites along the lower part of the Elbe River between the North Sea and Hamburg harbor. Areas shaded in light grey are municipalities. Sampling was done in autumn 2006.

Sediments were collected at eleven locations along the Elbe Estuary (Fig. 1) and included a reference location from a harbor basin in the area of the Elbe Estuary that was known to be more contaminated [27]. Sampling was carried out by the German Federal Institute of Hydrology (Koblenz, Germany). At each location, sediment was collected from the surface (0–20 cm) with a van Veen grab sampler and thoroughly homogenized in inert materials. Sediments were freeze-dried (Alpha 1–4, Christ, Osterode, Germany). Amounts of 20 g dw of the freeze-dried sediment (10 g dw for the reference site) were separately extracted with acetone (≥99.8%, Fluka, Buchs, Switzerland) for 12 h using standard reflux (Soxhlet) extractors at approximately ten cycles per hour according to the method detailed in Hollert et al, [28]. Extracts were reduced in volume to approximately 5 mL by use of a WB 2001 rotary evaporator (Heidolph, Kehlheim, Germany; 400 mbar, 36–38°C) and brought close to dryness under a gentle nitrogen stream. Residues from each sample were re-dissolved in 1 mL dimethyl sulfoxide (DMSO; ≥99.9%; Fluka) for bioassays or in 1 mL n-hexane (85%): toluene (15%) for chemical analysis, and stored at −20°C until testing. Empty extraction thimbles were subjected to the same extraction procedures in two parallel experiments, and served as process controls.

### 2.2 Multilayer fractionation

In order to identify unknown substances contributing to the dioxin-like potencies of whole extracts, a multilayer fractionation was performed to remove labile, acid-degradable compounds, such as non-persistent organics, including PAHs [29]. More persistent organic pollutants were separated via a sulfuric silica gel fractionation. The remaining fraction contained persistent compounds like PCDDs/PCDFs, PCNs and PCBs, some congeners of which are AhR agonists. The gels were prepared as follows: Silica gel 60 (70–230 mesh, Merck, Darmstadt, Germany) was washed with methanol (99.8%, Merck) and dichlormethane (99.99%, Fisher Scientific, Loughborough, UK) and activated at 130°C for 24 h. Both the sulfuric acid silica gels (40% and 20%) were made from activated silica gel and sulfuric acid (98%, Merck) and shaken until dry. For production of the KOH silica gel, 84 g KOH (≥86%, Fluka, Steinheim, Germany) were dissolved in 400 mL methanol, 150 g silica gel was added and the methanol was evaporated using a Laborota 4011-digital rotation evaporator (Heidolph, Kehlheim, Germany). Glass columns (15 mm inner diameter) were filled from the bottom up with 3 cm KOH silica gel, 0.5 cm neutral silica gel, 3 cm 40% sulfuric acid silica gel, 1.5 cm 20% sulfuric acid silica gel, 1 cm neutral silica gel, and 1 cm sodium sulfate (≥99%, Sigma-Aldrich, Steinheim, Germany). After the gels had been washed twice with *n*-hexane (>97%, Sigma-Aldrich), extracts were applied to the columns and eluted twice with *n*-hexane. Using a rotary evaporator, the volume of the eluates was reduced to 3 mL. This volume was then reduced to dryness under a nitrogen stream and then re-dissolved in 1 mL DMSO (99%, Grüssing, Filsum, Germany).

### 2.3 Ah receptor-mediated luciferase transactivation assay (H4IIE-*luc*)

H4IIE-*luc* cells were cultured in disposable petri plates (Corning, Corning NY, USA) under a humidified 95∶5 air∶CO_2_ atmosphere at 37°C. Dulbecco's Modified Eagle Medium (Sigma, St. Louis, MO, USA) supplemented with 10% fetal bovine serum was used for culturing (Sigma, USA). H4IIE-*luc* cells were obtained from Jac Aarts (University of Wageningen, The Nethderlands [19]). Cells were passaged when the cell layer had become confluent, and new cultures were started from frozen stocks, when cell age reached 30 passages. For testing, cells were trypsinized, diluted to approximately 7.5×10^4^ cells/mL and seeded into 60 interior wells of flat-bottom 96well microplates (ViewPlates, PerkinElmer, USA). Luciferase and protein assays was performed according to the method given in Koh et al. [7]. The 36 exterior wells were filled with 250 µl culture medium. Cells were incubated overnight to allow for cell attachment and then dosed. Test and control wells were dosed with 1 µl of solvent (DMSO) or the appropriate sample per mL culture medium (except 0.1 µl/mL for the reference site to insure that no cytotoxicity due to the greater toxicity of the reference site's sediment extract occurred). Blank wells received no solvent or sample. Dilutions of all samples, blanks and controls were tested in triplicate. Concentration-responses consisted of six concentrations prepared by 2-fold serial dilution from the maximum concentration tested. All exposures were incubated for duration of 72 h. To quantify luciferase activity, culture medium was removed and cells were rinsed with phosphate buffered saline (PBS). Cells were then treated with 50 µl 1 mmol L-1 Ca^2+^- and Mg^2+^-supplemented PBS and 50 µl LucLite™ reagent (PerkinElmer, Bosten, MA, USA). Plates were incubated for 10 min at 37°C and then scanned with a microplate-reading luminometer (OPTIMA POLARStar, Offenburg, Germany). Activity of luciferase, which was proportional to the potency of AhR agonists in the mixture, expressed as relative luminescence units (RLU), was expressed as a percentage of the maximum response observed for 2,3,7,8- TCDD, which could then be used to determine the concentration of TCDD equivalents.

### 2.4 Ah receptor-mediated RTL-W1 induction assay (RTL-W1)

Induction of 7-ethoxyresorufin *O*-deethylase (EROD) was measured by use of a CYP1A-expressing permanent fish liver cell line RTL-W1 isolated from rainbow trout (*Oncorhynchus mykiss*). RTL-W1 cells were provided from Drs. Niels C. Bols and Lucy Lee (University of Waterloo, Canada [20]) and were cultured at 20°C in 75 cm^2^ plastic culture flasks (TPP, Trasadingen, Switzerland) in Leibovitz medium supplemented with 10% fetal bovine serum (Sigma, Deisenhofen, Germany), 1% penicillin/streptomycin and 1% neomycin sulfate (Sigma). The assay was performed according to previously described methods [30], [31]. Prior to exposures, cells were seeded into 96-well plates (TPP) and allowed to grow to 100% confluence for 72 h. Subsequently, the medium was removed and the cells were exposed for 72 h to the samples diluted in medium to give a maximum concentration of 50 mg dw sediment equivalents (SEQ) per mL test medium for the crude sediment extracts and 200 mg dw SEQ/mL test medium for the multilayer fractions. The sample from the more polluted reference site was tested at a maximum concentration of 5 mg SEQ/mL for the crude sediment sample and at 20 mg dw SEQ/mL for the multilayer fraction. These different maximum concentrations were set to avoid cytotoxic effects. Concentration-responses consisted of eight concentrations prepared by 2-fold serial dilutions from the maximum concentration tested. The solvent content (DMSO) per well was less than 1%. A separate solvent control was tested with each sample. As the positive control, 2,3,7,8-TCDD (Promochem, Wesel, Germany) was serially diluted 2-fold to give a final concentration range of 3.13–100 pM on two separate rows of each plate. The growth medium was removed and the plates were frozen to −80°C to lyse the cells and to finally terminate the exposure. Deethylation of exogenous 7-ethoxyresorufin was initiated by adding 100 µl 1.2 umol L-1 7-ethoxyresorufin to each well and incubating in the dark at room temperature for 10 min before addition of 50 µl of 90 umol L-1 NADPH (Sigma-Aldrich). Plates were incubated for 10 min, and the reaction was stopped by adding 100 µl of 216 umol L-1 fluorescamine dissolved in acetonitrile. EROD activity was measured fluorometrically after another 15 min using a GENios plate reader (Tecan, Crailsheim, Germany; excitation 544 nm, emission 590 nm). Protein was determined fluorometrically (excitation 355 nm, emission 590 nm) using the fluorescamine method [32], [33]. Relative fluorescence units (RFU) were converted to pmol resorufin produced per min per mg protein (pmol/min/mg) by regression against the resorufin and protein curves.

### 2.5 Bioassay data analysis

Mean RLU or mean EROD activity (in pmol/min/mg) from replicate wells of the bioassay were converted to a percentage of the mean maximum responses observed for standard curves generated the same day (%-TCDD-max). The mean solvent control response was subtracted from both the sample and the 2,3,7,8-TCDD standard responses prior to conversion to percentages, to scale values from 0 to 100%-TCDD-max. Concentration-response curves were analyzed by nonlinear regression (GraphPad Prism 4, GraphPad Software Inc., La Jolla, CA, USA) using the classic sigmoid curve as the model equation.

The fitted curves were used for calculation of TEQ values in which usually two assumptions are made: To assure accurate estimates of TEQs, equal efficacy (maximum response achieved) and parallelism between the log-transformed 2,3,7,8-TCDD standard and unknown are required. In these studies, the efficacy of most of the tested extracts was less than that of 2,3,7,8-TCDD, i.e. mean RLU and EROD activities did not exceed 25%-TCDD-max. Because the units used to measure concentration are known for the standard curve, but not for the unknown environmental sample, it is impossible to test the assumption of parallelism of the concentration-response curves directly [34]. Thus, potency of the samples to induce a dioxin-like response in the assays was converted to 2,3,7,8-TCDD equivalents (TEQs) based on relating the EC_25TCDD_ value of each triplicate measurement of the extract, i.e. the concentration of sample that causes 25% of TCDD-max, to the EC_25_ of 2,3,7,8-TCDD (equation 1) [35]. Mean values and standard deviations of the TEQ values were calculated from independent triplicate assays.
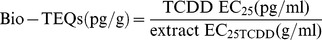
(1)


### 2.6 Chemical analysis

Quantification of the PAHs [36] was accomplished according to DIN ISO 18287:2006 [37]. As a sum parameter for the PAHs the list of the 16 EPA priority PAHs was used, which often is taken as representative for the measurement of this substance class in environmental samples. The sum parameter was calculated on the basis of the following PAHs: naphthalene, acenaphthylene, acenaphthene, fluorene, phenanthrene, anthracene, fluoranthene, pyrene, benzo[a]anthracene, chrysene, benzo[*b,j*]fluoranthene, benzo[*k*]fluoranthene, benzo[*a*]pyrene, dibenzo[*a,h*] anthracene, benzo[*g,h,i*]perylene, indeno [1,2,3-*cd*]pyrene. For the calculation of the sum parameter the measured concentrations of the single PAHs were added up for values above the limit of quantification. The chemical sediment analysis was performed by an accredited and certified laboratory GBA (Gesellschaft für Bioanalytik Hamburg mbH, Pinneberg, Germany). In preparation for chemical analysis, the sediment samples were freeze-dried and sieved with a 2 mm sieve. Then the samples were homogenized in a mortar (RM 200, Retsch GmbH, Germany) and in a flint mill (S1, Retsch GmbH, Germany) in analogy to ISO 11464. Afterwards the extraction was done by accelerated solvent extraction and the sample was cleaned up over an capillary column (DB-5). Analysis of PAHs was carried out by gas chromatography coupled to a mass-selective sensor (GC-MSD) with the Agilent 6890N GC and the Agilent 5973N MSD (Agilent Technologies GmbH, Böblingen, Germany). An automated sample injection system in splitless mode was used. A temperature and pressure controlled program ensured a constant gas flow. A method validation was carried out by the accredited laboratory. Also deuterated internal standards were measured (d8-naphthalene, d10-acenaphthene, d10-phenanthrene, d12-chrysene, d12-benzo[*g,h,i*]perylene). Hidden (blind) double determinations were commissioned for three sediment samples. The measurements did show a good correlation, except for one sample. Here the concentrations of benzo[*a*]pyrene, indeno [1,2,3-cd]pyrene, and benzo[*g,h,i*]perylene were close to the limits of quantification and deviated higher. The limits for quantification for the analyzed PAHs were 0.02 mg/kg dw for each PAH.

### 2.7 Chem-TEQ calculation

In order to determine the degree to which analytically measured PAHs accounted for the dioxin-like activity in both assays, PAH-TEQs were calculated on the basis of PAH-potencies relative to TCDD [38],as the sum of the product of the concentration of each of the 16 EPA-PAH multiplying by its respective bioassay-specific relative potency factor (ReP) from Bols et al. [22] and Machala et al. [23] for the RTL-W1 and H4IIE-*luc* cell-based assays, respectively (Equation 2).
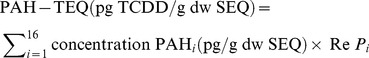
(2)


The PAH-TEQ, and “persistent”-TEQs derived from measuring the multilayer fraction values, were subtracted from the corresponding Bio-TEQ values to elucidate the percentage of the contribution of measured PAHs and of the acid-resistant organic pollutants, e.g. PCBs and PCDDs/PCDFs, to the total biological induction in each assay system.

## Results

### 3.1 AhR-mediated potency in the H4IIE-*luc*luciferase and RTL-W1 assays

**Figure 2:**
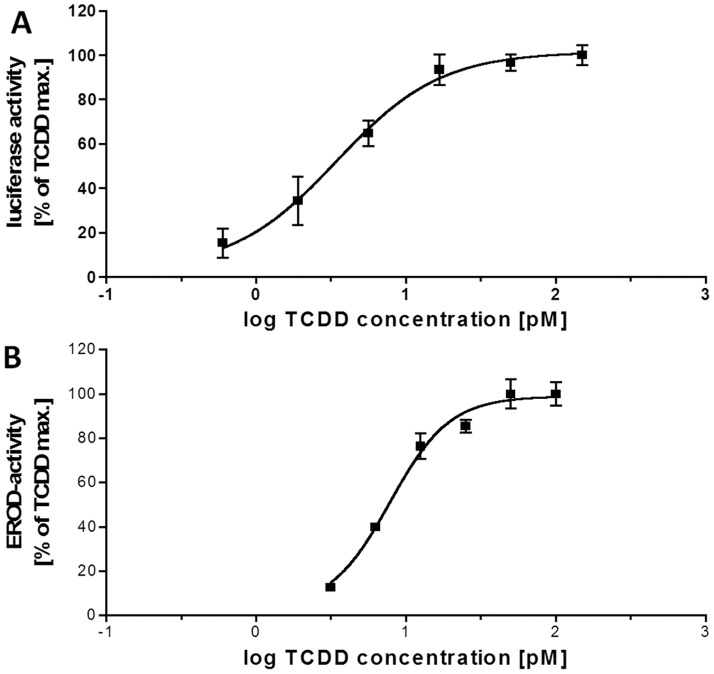
Dose-response curves of the luciferase assay (A) and the RTL-W1 assay (B) to the 2,3,7,8-tretrachlorodibenzo-*p*-dioxin (TCDD) standard.

Both bioassays responded similarly to TCDD (Fig. 2). The curve of the RTL-W1 assay was slightly steeper than that of the luciferase assay. The average EC25 of the 2,3,7,8-TCDD standard was 3.6±1.8 pM in the luciferase assay and 4.2±0.5 pM in the EROD assay with RTL-W1 throughout all performed assays, while the average EC50 was 9.9±4.3 and 7.9±2.3 pM, respectively. Only for the raw sediment extract from the reference site, induction efficiency exceeding 50% TCCD-max. was observed in the luciferase assay in all three replicate measurements. In the EROD assay with RTL-W1, none of the tested samples exceeded 50% TCCD-max. induction in all triplicate measurements. Calculation of TEQ values for raw extracts and multilayer fractions was therefore based on an effect-level of 25% TCDD-max. in both assays and for all samples.

**Figure 3:**
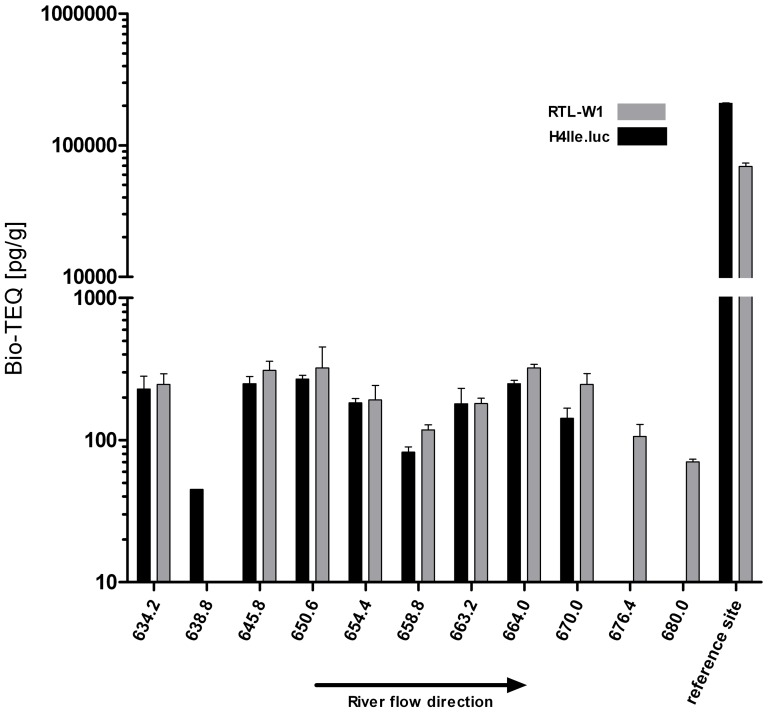
Dioxin-like activity of the crude sediment extracts in the RTL-W1 (grey bars; n = 3) and H4IIE.luc (black bars; n = 3) assays expressed as biological toxicity equivalents (Bio-TEQ; pg/g dw). If no grey or black bar is given, no Bio-TEQ was detectable. River kilometers and the known highly contaminated reference site are given on the x-axis.

Except for 3 locations, similar potencies of extracts were observed with both cell lines (Fig. 3). Almost all extracts caused a concentration-dependent increase in dioxin-like potency. The concentration–response curves varied in shape and in efficacy (maximum induction; details not shown). However, in most cases, concentration-response curves in the RTL-W1 assay were biphasic, most likely indicating onset of cytotoxicity at greater concentrations (sediment extract equivalents given in grams/mL). With exception of the more contaminated reference site and sample 638.8, potency of dioxin-like, AhR-mediated activation of luciferase activity, expressed as TCDD equivalent concentrations, measured with the H4IIE-*luc* assay were slightly less than those obtained with fish cells (Table 1). The greatest concentrations of Bio-TEQ were observed for extracts from locations at river kilometers 645.8, 650.6 and 664.0, as well as from the reference site (Table 1). The least potencies were measured in samples collected from sites at river kilometres 638.8, 676.4 and 680.0 (Table 1). No relationship between AhR agonist potencies of extracts and river kilometers were observed. There was a strong and significant correlation between the Bio-TEQs results of the RTL-W1 and H4IIE-*luc* assays (Spearman rank order coefficients of determination r^2^ = 0.96, p = 0.0003; except the reference site).

**Table 1:** Dioxin-like activity of the crude sediment extracts and the multilayer fractions in the RTL-W1 and H4IIE.luc assays expressed as biological toxicity equivalents (Bio-TEQ) in pg/g dw.

Sampling site	Crude sediment extracts		Multilayer fractions	
	*RTL-W1 assay*	*Luciferase assay*	*RTL-W1 assay*	*Luciferase assay*
634.2	246±80	228±90	23±2	n.d.
638.8	n.d.	44.9	n.d.	n.d.
645.8	308±89	248±55	n.d.	n.d.
650.6	322±183	268±31	15±12	n.d.
654.4	191±89	183±24	21.9	n.d.
658.8	117±18	82±10	n.d.	n.d.
663.2	180±29	180±89	89±52	111±69
664.0	321±33	248±26	n.d.	n.d.
670.0	246±82	142±44	21±3	n.d.
676.4	105±40	n.d.	8.9	n.d.
680.0	70.1±5	n.d.	n.d.	n.d.
ref. site	69,225±5,619	208,254±2,874	2,233±1,710	2,587±934

n.d. = not detectable/below detection limit.

Data are given as means of 3 replicates ± SD. If no standard deviation is given, Bio-TEQ values could only be calculated for one single triplicate.

When comparing concentrations of Bio-TEQ in multilayer fractions (containing acid-resistant organic pollutants like PCBs and PCDDs/PCDFs), responses measured by the H4IIE-*luc* assay were consistently less than those of the RTL-W1 assay, except for the samples from the reference site and river kilometer 663.2 (Table 1). These two sites were also the locations for which greatest dioxin-like potencies were observed in both assays. Extracts of sediments from all other locations either exhibited no or only small potencies for dioxin-like potency in either assay. The greatest AhR-mediated potency was observed with the H4IIE-*luc* assay for the fraction derived from the reference site (2,588 pg TEQ/g, dw). In comparison with crude extracts, the multilayer fractions in both test systems always had lesser potency than did the raw extract.

### 3.2 Concentrations of PAHs

There were significant differences in concentrations of PAHs in sediments among locations within the Elbe estuary (Table 2). The greatest sum of the concentations of the individual PAHs (∑PAHs) (US EPA 610) was observed in sediment from the reference harbor site (509.4 µg/g dw). In sediments from the Elbe estuary, the greatest concentrations of ∑PAHs were found in sediments from sites 676.4 (0.906 µg/g dw) and 645.8 (0.871 µg/g dw). For sites 638.8 and 680.0, concentrations of all 16 PAHs were consistently less than the limit of quantification (<0.02 µg/g dw). Cumulative concentrations of the 16 PAHs for the other sites were between 0.26 and 0.769 µg/g dw. The greatest individual concentrations were measured for fluoranthene (0.06–0.17 µg/g dw), pyrene (0.05–0.14 µg/g dw), and benzo(*b*)fluoranthene (0.036–0.1 µg/g dw).

**Table 2:** Concentrations of the 16 US EPA polycyclic aromatic hydrocarbons PAH in sediment samples from the river Elbe estuary expressed as micrograms per gram of sediment n. n. = no number available.

Sampling site	634.2	638.8	645.8	650.6	654.4	658.8	663.2	664.0	670.0	676.4	680	ref. site
Naphthaline	<0.020	<0.020	0.022	0.025	<0.020	<0.020	<0.020	<0.020	<0.020	<0.020	<0.020	7.1
Acenaphthylene	<0.020	<0.020	<0.020	<0.020	<0.020	<0.020	<0.020	<0.020	<0.020	<0.020	<0.020	<0.5
Acenaphthene	<0.020	<0.020	<0.020	<0.020	<0.020	<0.020	<0.020	<0.020	<0.020	<0.020	<0.020	7.9
Fluorene	<0.020	<0.020	<0.020	<0.020	<0.020	<0.020	<0.020	<0.020	<0.020	<0.020	<0.020	11
Phenanthrene	0.065	<0.020	0.078	0.073	0.056	0.029	0.025	0.044	0.04	0.077	<0.020	7
Anthracene	<0.020	<0.020	0.021	0.02	<0.020	<0.020	<0.020	<0.020	<0.020	<0.020	<0.020	<0.1
Fluoranthene	0.13	<0.020	0.16	0.14	0.14	0.06	0.061	0.11	0.079	0.17	<0.020	114
Pyrene	0.11	<0.020	0.14	0.12	0.12	0.05	0.056	0.092	0.07	0.13	<0.020	77
Benz*a*anthracene	0.044	<0.020	0.066	0.059	0.059	0.029	<0.020	0.038	0.029	0.075	<0.020	46
Chrysene	0.052	<0.020	0.083	0.064	0.071	0.036	0.031	0.046	0.036	0.11	<0.020	49
Benzo*b*fluoranthene	0.073	<0.020	0.08	0.091	0.073	0.041	0.036	0.043	0.054	0.1	<0.020	52
Benzo*k*fluoranthene	0.045	<0.020	0.05	0.041	0.043	0.025	0.025	0.032	0.03	0.062	<0.020	23
Benzo*a*pyrene	0.055	<0.020	0.074	0.061	0.061	0.036	0.026	0.043	0.04	0.079	<0.020	46
Indeno1,2,3-*cd*pyrene	0.037	<0.020	0.043	0.033	0.032	0.021	<0.020	0.022	0.032	0.05	<0.020	33
Dibenz*a,h*anthracene	<0.020	<0.020	<0.020	<0.020	<0.020	<0.020	<0.020	<0.020	<0.020	<0.020	<0.020	3.4
Benzo*g,h,i*perylene	0.045	<0.020	0.054	0.042	0.039	0.022	<0.020	0.026	0.036	0.053	<0.020	33
**Sum PAH EPA**	**0.656**	**n.n.**	**0.871**	**0.769**	**0.694**	**0.349**	**0.26**	**0.496**	**0.446**	**0.906**	**n.n.**	**509.4**

### 3.3 Correlation between Bio-TEQs and chemical analyses

**Figure 4:**
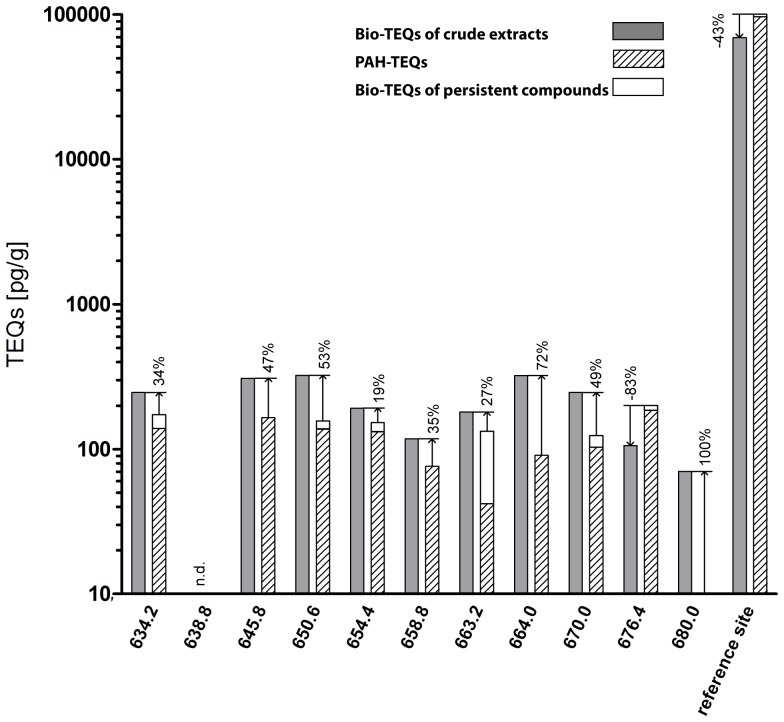
Comparison of the total biological response in the RTL-W1 assay (Bio-TEQs) of crude sediment extracts, the calculated contribution of the measured PAH toxic equivalents (TEQs) and the TEQs of non-acid-degradable compounds (e.g. PCB and PCDD/PCDF) out of the biological response to the multilayer fraction. The unknown portion (in percent) of the overall activities is given in regard to the Bio-TEQs of the crude extracts. PAH-TEQs were calculated using the relative potency factors taken from Bols et al. 1999 and are given in pg/g dw [22]. (n.d. = not detectable).

Concentrations of Chem-TEQs were compared to those of Bio-TEQs to identify the contribution of the measured 16 US EPA PAHs and of the fraction of non-acid-degradable compounds, such as PCDD/Fs, PCBs and PCNs). For most of the crude extracts of sediments measured in the RTL-W1 assay, the proportion of the Bio-TEQ in excess of the proportion explained by the Chem-TEQ ranged between 53% for the sample collected at site 650.6-S and 19% for the sample collected at site 654.4-P (Fig. 4). For sampling sites 664.0 and 680.0, the fractions of the proportion of Bio-TEQ that remained unexplained by concentrations of Chem-TEQs were 72% and 100%, respectively. In the case of site 676.4 and the reference harbor site, the contribution of PAHs and persistent compounds to the biological response exceeded 100%. Overall, the 16 EPA-PAHs contributed the greatest proportion of Chem-TEQ and in most cases represented a significant proportion of the AhR-mediated potency of crude extracts of sediments in the RTL-W1 assay. Persistent compounds contained in the multilayer fractions represented only a small proportion of the total AhR-mediated potency except for the sample collected at site 663.2. Here, 49.6% of the Bio-TEQs of the crude extracts could be explained by the presence of chemicals in the fraction containing persistent compounds. No correlation between Bio-TEQs and river kilometers was observed.

**Figure 5:**
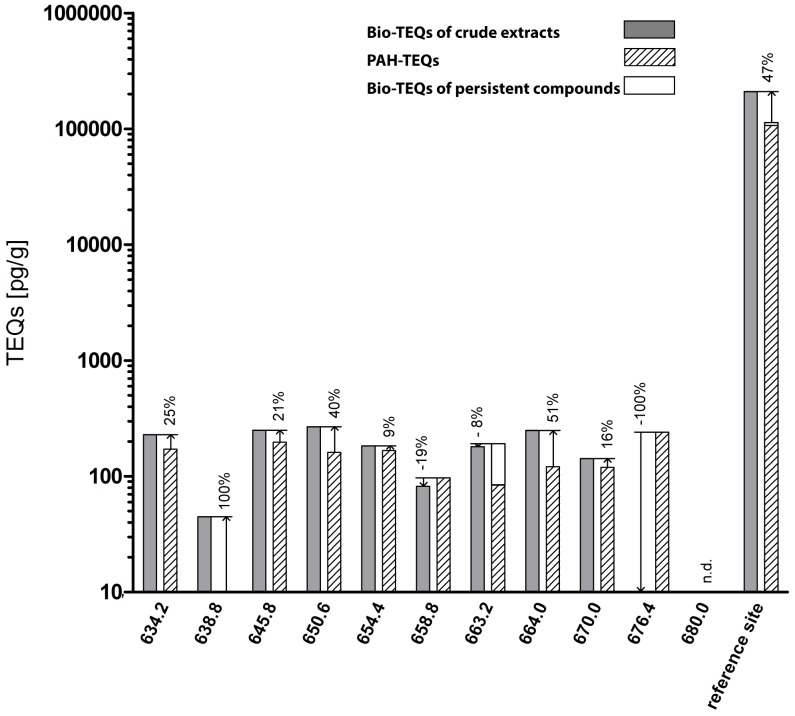
Comparison of the total biological response in the H4IIE.luc assay (Bio-TEQs) of crude sediment extracts, the calculated contribution of the measured PAH toxic equivalents (TEQs) and the TEQs of non-acid-degradable compounds (e.g. PCB and PCDD/PCDF) out of the biological response to the multilayer fraction. The unknown portion (in percent) of the overall activities is given in regard to the Bio-TEQs of the crude extracts. PAH-TEQs were calculated using the relative potency factors taken from Machala et al. 2001 and are given in pg/g dw. (n.d. = not detectable).

The proportion of the Bio-TEQ that was not accounted for by the concentrations of PAH-TEQ was similar in both assays (Fig. 5). The unknown portion of the Bio-TEQs was between 9 and 51% for extracts of sediments from most locations including the reference harbor. At only one location, river kilometer 638.8, could none of the Bio-TEQ in the raw extract be explained with the chemical data. Similar to the results obtained with the RTL-W1 assay at site 676.4, the Chem-TEQs did not explain any of the Bio-TEQ. For the extract of sediment from site 663.2, 61% of the Bio-TEQ could be attributed to TEQs of persistent compounds. In contrast, to all other sites, the persistent compounds in the multilayer fractionation contributed little to the Bio-TEQ determined by use of the H4IIE-*luc* assay (4–11% for all sites except for 44% at site 663.2-S).

The majority of Bio-TEQ measured in extracts of sediments from the Elbe River by both assays could be accounted for by PAHs. In contrast, the majority of the AhR-mediated potency of crude extracts of sediments from site 663.2 was explained by TEQs of persistent compounds. The percentage of unidentified contributors to Bio-TEQs of crude extracts was twice as great for the RTL-W1 assay than the H4IIE-*luc* system (Figs. 4 and 5).

## Discussion

The results of the present study demonstrate the applicability of two cell-based bioassays to characterize dioxin-like activities in crude and fractionated extracts of sediments collected in the Elbe Estuary. Almost all crude extracts of sediments from the Elbe Estuary tested, exhibited AhR-mediated potencies in both the H4IIE-*luc* and luciferase and RTL-W1 assays; however, with assay-specific differences. In general, RTL-W1 cells exhibited slightly greater induction than did the H4IIE-*luc* cells. Furthermore, the percentage of unidentified contributors to concentrations of Bio-TEQs of crude extracts was greater for the RTL-W1 assay. Differences in magnitude of response proportion of unidentified contributors between these two cell lines might be due to species-specific differences in responses to AhR agonists, different endpoint measurements and different exposure times [6], [9], [21], [30], [39]. As previously demonstrated, a mixture of the 16 US-EPA PAHs was more potent in the RTL-W1 assay than in the luciferase system [9]. This is consistent with the results observed in this study in which greater magnitude or response was observed in RTL-W1 cells compared to the H4IIE-*luc* cells considering the relatively great proportion of the Bio-TEQ that could be attributed to these PAHs. Exposure to mixtures of chemicals in extracts of sediments might affect the two test system differentially due to interactions among the constituents, including both AhR-active and -inactive congeners [19]. Nevertheless, the strong correlation between the results obtained with both assays demonstrated the suitability of both tests to characterize the exposure to dioxin-like chemicals in natural sediments. This result is also consistent with the correlation shown by Keiter at al. [21] for induction of AhR-mediated potency after exposure of H4L1.1c4 (DR-CALUX® [40] and RTL-W1 (RTL-W1) cells to crude extracts of sediments from the upper Danube River, Germany. Likewise, with regard to the response to TCDD as a single substance, both assays showed similar sensitivities across tested concentrations, which is consistent with the results or previous studies [9].

Several investigations on European, Asian and South-American streams revealed different concentrations of Bio-TEQ determined either by the RTL-W1 or H4IIE-*luc* assays for surface extracts of sediments from rivers under normal flow conditions [2], [21], [38], [41]. For example, surface sediments from the Danube River contained concentrations of Bio-TEQ that resulted in these sediments being classified as “highly contaminated” and were approximately 10-fold greater than those measured in sediments from the Elbe Estuary during this study (Keiter et al. [21]). Another study by Heimann et al. [38] found even greater concentrations of Bio-TEQ ranging from 3,620 to 7,920 pg/g) for sediments collected from an oxbow lake from the River Rhine. Sediments from the Tietê River, Brazil, contained concentrations of Bio-TEQ that were comparable to those reported for the highly contaminated reference site in the Elbe Estuary during the present study [41]. In a study with sediments from the Hyeongsan River, Korea, using the H4IIE-*luc* Koh et al. [7] measured concentrations of Bio-TEQs ranging from 0.01 to 1520 pg/g dw for what was characterized as a “highly polluted” site. Fractionation techniques as well as chemical analysis allowed the identification of PCDD/Fs as the major source of total Bio-TEQs in extracts of sediments from the Hyeongsan River while PAHs and PCBs accounted for less than 20% and 15% of the total Bio-TEQ, respectively. For the majority of locations investigated in the present study, POPs and mainly PAHs were identified as the major contributors to the Bio-TEQs in both bioassays. This corresponds well with the constant input of PAHs and HAHs bound to SPM from the upper course of the Elbe River [12]. Such large contributions of priority PAHs and POPs to the overall biological AhR-mediated potency had rarely been reported to date, and is in contrast to a number of similar studies with sediments collected from the Elbe, Tietê, and Danube Rivers, where only small fractions of AhR-mediated potency could be attributed to priority PAHs or POPs [2], [21], [41]. However, albeit less than the proportion observed in these previous studies, a significant fraction of the AhR-mediated potency measured in the present study could not be attributed to PAHs. Other non-priority pollutants have been shown to mediate AhR-mediated potency [2], [42]. Recently, heterocyclic aromatic compounds have been demonstrated to be AhR agonists [43], and Brack et al. [44] demonstrated that PCNs contributed as much as 10% of the total AhR-mediated potency of sediments from the Elbe River upstream of the sites investigated in the present study. As sediments are relocated during flood events [42], [45], it is likely that heterocyclic aromatic compounds are transported along the course of the river and can contribute to the overall AhR-mediated potency in the Elbe Estuary. Additionally, plant-derived materials such as humic and fulvic acids that are found in soils and sediments commonly contain AhR-ligands or products that can be converted into AhR-ligands [46], [47], [48], [49], and, as such, they can contribute to the proportion of AhR-mediated potency measured in the present study that could not be attributed to priority PAHs or POPs.

The 16 US-EPA PAHs were identified as major contributors of Bio-TEQs in both assays and are known to represent a major source of organic pollution in sediments of the Elbe Estuary. Concentrations of ∑PAHs in sediments collected from locations along the Elbe River estuary did not exceed 1 µg/g dw for the 16 US-EPS PAHs in a single sample. To classify hot spots, rank contaminated sites and trigger more detailed studies on site-specific effects in aquatic communities, classification schemes and recommendations have been developed for environmental authorities dealing with sediment quality assessment. Based on these classification schemes, regulatory action can be triggered and remediation objectives for dredged sediments can be established. According to the classification scheme of the ARGE-Elbe [13], all sampling sites in the present study would have been categorized as level I, indicating that they do meet the quality goal for sediments given by the ARGE-Elbe. However, no assumptions can be made for potential adverse effects on ecosystem health from the measured PAH concentrations in sediments of the Elbe Estuary. Studies conducted in the tidal Potomac River watershed, USA, and the Tockahoe River, MD, USA, where the latter of which served as an unpolluted reference site, could not establish a significant cause-effect linkage between pollution status and fish health even though the concentration of ∑PAHs bound in sediments was 10- to 100-fold greater than that observed in the Elbe Estuary during this study [50]. In addition, if compared to results of studies that assessed concentrations of dioxin-like chemicals, particularly PAHs, in larger European streams the concentration of ∑PAHs in sediments of the Elbe Estuary appear relatively small. Specifically, dioxin-like potentials measured in the Elbe Estuary were approximately 10-fold less than those reported for the Rhine, Danube and upper part of the Elbe Rivers [21], [42], [51]. Based on an assessment of rates of transport of sediments and SPM between the North Sea and the Elbe Estuary a dilution effect has to be assumed for pollutants originating from the upper part of the Elbe River towards the North Sea [11]. Additionally a dynamic system with changing physico-chemical and hydraulic properties has to be assumed for the Elbe Estuary. Even additional loading during a historically large flood event in 2002 caused only a slight temporal increase in contaminant concentrations in sediments of the estuary. Comparison with data taken before the flood event indicates that concentration levels returned to pre-flood levels within a short time [52], [53].

In contrast, the sum concentrations of priority PAHs in the sample from a known highly contaminated reference harbor site in the catchment area of the Elbe Estuary were 510 µg ∑PAHs/g dw (class V by the ARGE-Elbe classification system), which indicated that sediments from this small disused hazardous harbor basin did not reach the quality goal of 1 to 4 µg ∑PAH/g dw.

Results of standardised assays with marine and freshwater species applied in a study in autumn 2006, including luminescent bacteria test with *Vibrio fischeri* (DIN EN ISO 11348-3), freshwater algae test with *Desmodesmus subspicatus* (DIN 38412-33), acute *Daphnia* toxicity test (DIN 38412-30), marine algae test with *Phaedactylum tricomutum* (DIN EN ISO 10253), and amphipod toxicity test with *Corophium volutator* (DIN EN ISO 16712) revealed toxicity for few of the 48 locations [11]. The few locations at which sediments were determined to be toxic were ranked as “very low” or “low” toxicological hazard potential, mainly based on toxicity to algae. The results of the *in vitro* assays utilized during the present study which are reported here indicated low to moderate concentrations of AhR agonists in sediments from locations 645.8, 650.6 and 664.0. Based on the concentrations of Bio-TEQ in extracts from sediments at these locations as well as the relatively small concentrations at other locations sediments in the Elbe Estuary were classified as having a little ecotoxicological hazard. The mechanism-specific data obtained in the present study for dioxin like compounds that are agonists of the AhR do not pose significant risks to fish due to the exposure to the more labile compounds such as PAHs or other more recalcitrant dioxin-like chemicals. However, it can be concluded that the overall ecotoxicological potential is only partially reflected in the results reported here. Alternatively, the AhR-mediated potencies could not be completely explained by chemical analysis of priority pollutants, which lead to the conclusion that other unknown non-priority chemicals were likely to have contributed to the observed effects. Also, as shown by a number of other studies [21], [54], [55], [56], [57], objective evaluation of risks posed by contaminants associated with sediments characterized by exposure to mixtures of compounds such as those analyzed in this study requires a holistic assessment approach including measurement of a number of different, mechanistically driven parameters (such as mutagenic, genotoxic, teratogenic, and estrogen-receptor like responses) along with a statistical evaluation approaches that are able to deal with complex data of ecotoxicological risk assessment (e.g. fuzzy-logic or weight of evidence approaches) [13], [58], [59], [60]. Thus, a potential impact of these possibly toxic and unknown non-priority chemicals on fish health cannot be fully ruled out. However, since the bioassays used in the present study integrate a range of chemicals with varying physical and chemical properties and different potential of metabolic alteration, TEQ values are not directly suitable to predict the movement of compounds between different environmental compartments, e.g. in bioaccumulation studies [7], [25], [26]. The combination of bioassays with chemical analyses as used in, e.g., effect-directed analysis, is a powerful tool to identify the unknown substances causing the effect in bioassays [1], [61]. “Another promising approach is the interdisciplinary combination of methods from hydraulic engineering and ecotoxicology in so-called “hydrotoxic” investigations, which can provide information on the toxicological impact of sediment-borne contaminants under realistic exposure conditions [62].

When considering potential causes of the decrease in standing stocks of fishes a number of factors can contribute to altered or even declining sizes of fish populations, not only chemical contamination has to be taken into account, but also other factors, e.g. habitat alteration [63] and primary water quality parameters such as oxygen concentration [64]. Hydraulic engineering during the last 150 years has changed the estuary tremendously: Habitats like backwaters and shallow waters have disappeared, the channel depth has been dredged from originally approximately 3.5 to 14.9 meters, and the tidal range has increased [11], [65]. Along with this, concentrations of oxygen less than the critical threshold of 3 mg O_2_/L for survival of fish have been reported during the summer season in the Elbe Estuary. Seasons of low oxygen concentrations have been less frequently since the 1990s, but may still occur [11], [14]. In conclusion, altered or declined fish populations in the Elbe Estuary are likely due to a number of factors with habitat alteration and physicochemical parameters being the most relevant ones while chemical pollution probably represents a secondary contributor. Additionally, this is one of the seldom reports on presumptive low chemical contribution to overall ecosystem stressors. Effect directed analysis may now help to reveal the remaining chemical contributors to the unknown portion of overall biological AhR-mediated potency.

## References

[1] Brack W (2003) Effect-directed analysis: a promising tool for the identification of organic toxicants in complex mixtures? Analytical and bioanalytical chemistry 377: 397–407. 12904950 10.1007/s00216-003-2139-z

[2] Brack W, Schirmer K, Erdinger L, Hollert H (2005) Effect-directed analysis of mutagens and ethoxyresorufin-O-deethylase inducers in aquatic sediments. Environmental Toxicology and Chemistry 24: 2445–2458. 16268146 10.1897/05-078r.1

[3] Villeneuve DL, Kannan K, Khim JS, Falandysz J, Nikiforov VA, et al (2000) Relative Potencies of Individual Polychlorinated Naphthalenes to Induce Dioxin-Like Responses in Fish and Mammalian In Vitro Bioassays. Archives of environmental contamination and toxicology 39: 273–281. 10948276 10.1007/s002440010105

[4] Barron MG, Carls MG, Heintz R, Rice SD (2004) Evaluation of Fish Early Life-Stage Toxicity Models of Chronic Embryonic Exposures to Complex Polycyclic Aromatic Hydrocarbon Mixtures. Toxicological Sciences 78: 60–67. 14691206 10.1093/toxsci/kfh051

[5] King-Heiden TC, Mehta V, Xiong KM, Lanham KA, Antkiewicz DS, et al (2011) Reproductive and developmental toxicity of dioxin in fish. Molecular and Cellular Endocrinology 354: 121–138. 21958697 10.1016/j.mce.2011.09.027 PMC3306500

[6] Villeneuve D, Khim J, Kannan K, Giesy J (2002) Relative potencies of individual polycyclic aromatic hydrocarbons to induce dioxinlike and estrogenic responses in three cell lines. Environmental toxicology 17: 128–137. 11979591 10.1002/tox.10041

[7] Koh CH, Khim J, Kannan K, Villeneuve D, Senthilkumar K, et al (2004) Polychlorinated dibenzo-p-dioxins (PCDDs), dibenzofurans (PCDFs), biphenyls (PCBs), and polycyclic aromatic hydrocarbons (PAHs) and 2, 3, 7, 8-TCDD equivalents (TEQs) in sediment from the Hyeongsan River, Korea. Environmental Pollution 132: 489–501. 15325465 10.1016/j.envpol.2004.05.001

[8] Kannan K, Imagawa T, Blankenship AL, Giesy JP (1998) Isomer-Specific Analysis and Toxic Evaluation of Polychlorinated Naphthalenes in Soil, Sediment, and Biota Collected near the Site of a Former Chlor-Alkali Plant. Environmental science & technology 32: 2507–2514.

[9] Villeneuve D, Khim J, Kannan K, Giesy J (2001) In vitro response of fish and mammalian cells to complex mixtures of polychlorinated naphthalenes, polychlorinated biphenyls, and polycyclic aromatic hydrocarbons. Aquatic toxicology 54: 125–141. 11451431 10.1016/s0166-445x(00)00171-5

[10] Van den Berg M, Birnbaum LS, Denison M, De Vito M, Farland W, et al (2006) The 2005 World Health Organization reevaluation of human and Mammalian toxic equivalency factors for dioxins and dioxin-like compounds. Toxicol Sci 93: 223–241. 16829543 10.1093/toxsci/kfl055 PMC2290740

[11] BfG (2008) WSV-Sedimentmanagement Tideelbe – Strategien und Potentiale – eine Systemstudie. Ökologische Auswirkungen der Umlagerung von Wedeler Baggergut.: Bundesanstalt für Gewässerkunde, Koblenz, Germany.

[12] Heise S, Claus E, Heininger P, Krämer T, Krüger F, et al (2005) Studie zur Schadstoffbelastung der Sedimente im Elbeeinzugsgebiet. Hamburg Port Authority

[13] Ahlf W, Hollert H, Neumann-Hensel H, Ricking M (2002) A guidance for the assessment and evaluation of sediment quality a German Approach based on ecotoxicological and chemical measurements. Journal of Soils and Sediments 2: 37–42.

[14] FGG-Elbe (2012) Fachinformationssystem (FIS) der FGG Elbe.

[15] Wetzel MA, Wahrendorf DS, von der Ohe PC (2013) Sediment pollution in the Elbe estuary and its potential toxicity at different trophic levels. The Science of the total environment 449: 199–207. 23428749 10.1016/j.scitotenv.2013.01.016

[16] Gaumert T (2002) Historischer Zustand der Elbe bei Hamburg. Flussgebietsgemeinschaft Elbe (FGG Elbe).

[17] Cameron P, Berg J, von Westernhagen H, Detlefsen V (1990) Missbildungen bei Fischembryonen der südlichen Nordsee. In: Lozan J, Lenz W, Rachor E, Watermann B, von Waeternhagen H, editors. Warnsignale aus der Nordsee. Berlin/Hamburg: Parey. pp. 281–294.

[18] Hilscherova K, Machala M, Kannan K, Blankenship AL, Giesy JP (2000) Cell Bioassays for Detection of Aryl Hydrocarbon (AhR) and Estrogen Receptor (ER) Mediated Activity in Environmental Samples. Environ Sci Pollut Res 7: 159–171. 10.1065/espr2000.02.017 19104878

[19] Sanderson JT, Aarts J, Brouwer A, Froese KL, Denison MS, et al (1996) Comparison of Ah receptor-mediated luciferase and ethoxyresorufin-O-deethylase induction in H4IIE cells: implications for their use as bioanalytical tools for the detection of polyhalogenated aromatic hydrocarbons. Toxicology and applied pharmacology 137: 316–325. 8661358 10.1006/taap.1996.0086

[20] Lee LEJ, Clemons JH, Bechtel DG, Caldwell SJ, Han KB, et al (1993) Development and characterization of a rainbow trout liver cell line expressing cytochrome P450-dependent monooxygenase activity. Cell Biology and Toxicology 9: 279–294. 8299006 10.1007/BF00755606

[21] Keiter S, Grund S, van Bavel B, Hagberg J, Engwall M, et al (2008) Activities and identification of aryl hydrocarbon receptor agonists in sediments from the Danube river. Analytical and bioanalytical chemistry 390: 2009–2019. 17938895 10.1007/s00216-007-1652-x

[22] Bols N, Schirmer K, Joyce E, Dixon D, Greenberg B, et al (1999) Ability of polycyclic aromatic hydrocarbons to induce 7-ethoxyresorufin-o-deethylase activity in a trout liver cell line. Ecotoxicology and Environmental Safety 44: 118–128. 10499998 10.1006/eesa.1999.1808

[23] Machala M, Vondrácek J, Bláha L, Ciganek M, Neca J (2001) Aryl hydrocarbon receptor-mediated activity of mutagenic polycyclic aromatic hydrocarbons determined using in vitro reporter gene assay. Mutation Research/Genetic Toxicology and Environmental Mutagenesis 497: 49–62. 10.1016/s1383-5718(01)00240-6 11525907

[24] Willett K, Gardinali P, Sericano J, Wade T, Safe S (1997) Characterization of the H4IIE rat hepatoma cell bioassay for evaluation of environmental samples containing polynuclear aromatic hydrocarbons (PAHs). Archives of environmental contamination and toxicology 32: 442–448. 9175514 10.1007/s002449900211

[25] Khim JS, Kannan K, Villeneuve DL, Koh CH, Giesy JP (1999) Characterization of TCDD- and Estrogen-like Activity in Sediment from Masan Bay, Korea Using: 2. In vitro Gene Expression Assays. Environ Sci Technol 33: 4206–4211.

[26] Khim JS, Kannan K, Villeneuve DL, Koh CH, Giesy JP (1999) Characterization and Distribution of Trace Organic Contaminants in Sediment from Masan Bay, Korea: 1. Instrumental Analyses. Environ Sci Technol 33: 4199–4205.

[27] Gotz R, Schumacher E, Kjeller LO, Bergqvist PA, Rappe C (1990) Polychlorinated Dibenzo-Para-Dioxins and Polychlorinated Dibenzofurans in Sediments and Fish in the Harbor of Hamburg. Chemosphere 20: 51–73.

[28] Hollert H, Dürr M, Olsman H, Halldin K, van Bavel B, et al (2002) Biological and chemical determination of dioxin-like compounds in sediments by means of a sediment triad approach in the catchment area of the River Neckar. Ecotoxicology 11: 323–336. 12463678 10.1023/a:1020549103898

[29] Wölz J, Engwall M, Maletz S, Olsman Takner H, van Bavel B, et al (2008) Changes in toxicity and Ah receptor agonist activity of suspended particulate matter during flood events at the rivers Neckar and Rhine - a mass balance approach using in vitro methods and chemical analysis. Environ Sci Pollut Res Int 15: 536–553. 18936997 10.1007/s11356-008-0056-6

[30] Olsman H, Engwall M, Kammann U, Klempt M, Otte J, et al (2007) Relative differences in aryl hydrocarbon receptor-mediated response for 18 polybrominated and mixed halogenated dibenzo-P-dioxins and-furans in cell lines from four different species. Environmental Toxicology and Chemistry 26: 2448–2454. 17941736 10.1897/07-004R.1

[31] Gustavsson LK, Klee N, Olsman H, Hollert H, Engwall M (2004) Fate of Ah receptor agonists during biological treatment of an industrial sludge containing explosives and pharmaceutical residues. Environ Sci Pollut Res Int 11: 379–387. 15603527 10.1007/BF02979656

[32] Brunström B, Halldin K (1998) EROD induction by environmental contaminants in avian embryo livers. Comparative biochemistry and physiology Part C, Pharmacology, toxicology & endocrinology 121: 213–219. 10.1016/s0742-8413(98)10042-7 9972463

[33] Lorenzen A, Kennedy SW (1993) A fluorescence-based protein assay for use with a microplate reader. Analytical biochemistry 214: 346–348. 8250247 10.1006/abio.1993.1504

[34] Villeneuve DL, Blankenship AL, Giesy JP (2000) Derivation and application of relative potency estimates based on in vitro bioassay results. Environmental Toxicology and Chemistry 19: 2835–2843.

[35] Brack W, Segner H, Möder M, Schüürmann G (2000) Fixed-effect-level toxicity equivalents—a suitable parameter for assessing ethoxyresorufin-O-deethylase induction potency in complex environmental samples. Environmental Toxicology and Chemistry 19: 2493–2501.

[36] US-EPA (1982) Laboratory Test Protocol Number 610.

[37] DIN-ISO-16703S (2005) Soil quality - Determination of content of hydrocarbon in the range C10 to C40 by gas chromatography (ISO 16703:2004); EN ISO 16703.

[38] Heimann W, Sylvester M, Seiler TB, Hollert H, Schulz R (2011) Sediment toxicity in a connected oxbow lake of the Upper Rhine (Germany): EROD induction in fish cells. Journal of Soils and Sediments 11: 1279–1291.

[39] Birnbaum LS, Staskal DF, Diliberto JJ (2003) Health effects of polybrominated dibenzo-p-dioxins (PBDDs) and dibenzofurans (PBDFs). Environment international 29: 855–860. 12850101 10.1016/S0160-4120(03)00106-5

[40] Murk A, Legler J, Denison M, Giesy J, Van de Guchte C, et al (1996) Chemical-activated luciferase gene expression (CALUX): a novel in vitro bioassay for Ah receptor active compounds in sediments and pore water. Toxicological Sciences 33: 149–160. 10.1006/faat.1996.0152 8812260

[41] Rocha PS, Azab E, Schmidt B, Storch V, Hollert H, et al (2010) Changes in toxicity and dioxin-like activity of sediments from the Tietę River (São Paulo, Brazil). Ecotoxicology and Environmental Safety 73: 550–558. 20074803 10.1016/j.ecoenv.2009.12.017

[42] Wölz J, Brack W, Moehlenkamp C, Claus E, Braunbeck T, et al (2010) Effect-directed analysis of Ah receptor-mediated activities caused by PAHs in suspended particulate matter sampled in flood events. Science of the Total Environment 408: 3327–3333. 20417549 10.1016/j.scitotenv.2010.03.029

[43] Hinger G, Brinkmann M, Bluhm K, Sagner A, Takner H, et al (2011) Some heterocyclic aromatic compounds are Ah receptor agonists in the DR-CALUX assay and the EROD assay with RTL-W1 cells. Environmental Science and Pollution Research 18: 1297–1304. 21431309 10.1007/s11356-011-0483-7

[44] Brack W, Bláha L, Giesy JP, Grote M, Moeder M, et al (2008) Polychlorinated naphthalenes and other dioxin-like compounds in Elbe River sediments. Environmental Toxicology and Chemistry 27: 519–528. 17973563 10.1897/07-400.1

[45] Reincke H (2003) Hochwasser August 2002: Einfluss auf die Gewässergüte der Elbe. Arbeitsgemeinschaft für die Reinhaltung der Elbe

[46] Denison MS, Nagy SR (2003) Activation of the aryl hydrocarbon receptor by structurally diverse exogenous and endogenous chemicals. Annu Rev Pharmacol Toxicol 43: 309–334. 12540743 10.1146/annurev.pharmtox.43.100901.135828

[47] Doostdar H, Burke MD, Mayer RT (2000) Bioflavonoids: selective substrates and inhibitors for cytochrome P450 CYP1A and CYP1B1. Toxicology 144: 31–38. 10781868 10.1016/s0300-483x(99)00215-2

[48] Huuskonen SE, Hahn ME, Lindstrom-Seppa P (1998) A fish hepatoma cell line (PLHC-1) as a tool to study cytotoxicity and CYP1A induction properties of cellulose and wood chip extracts. Chemosphere 36: 2921–2932. 9734273 10.1016/s0045-6535(97)10248-x

[49] Bittner M, Janošek J, Hilscherová K, Giesy JP, Holoubek I, et al (2006) Activation of Ah Receptor by Pure Humic Acids. Environ Toxicol 21: 338–342. 16841312 10.1002/tox.20185

[50] Pinkney AE, Harshbarger JC, May EB, Melancon MJ (2001) Tumor prevalence and biomarkers of exposure in brown bullheads (Ameiurus nebulosus) from the tidal Potomac River, USA, watershed. Environ Toxicol Chem 20: 1196–1205. 11392129

[51] Umlauf G, Stachel B, Mariani G, Götz R (2011) Dioxins and PCBs in solid matter from the River Elbe, its tributaries and the North Sea (longitudinal profile, 2008). JRC Technical and Scientific Reports, European Union EUR 24766 EN.

[52] Stachel B, Christoph EH, Gotz R, Herrmann T, Kruger F, et al (2006) Contamination of the alluvial plain, feeding-stuffs and foodstuffs with polychlorinated dibenzo-p-dioxins, polychlorinated dibenzofurans (PCDD/Fs), dioxin-like polychlorinated biphenyls (DL-PCBs) and mercury from the River Elbe in the light of the flood event in August 2002. The Science of the total environment 364: 96–112. 16199077 10.1016/j.scitotenv.2005.07.004

[53] Stachel B, Jantzen E, Knoth W, Kruger F, Lepom P, et al (2005) The Elbe flood in August 2002–organic contaminants in sediment samples taken after the flood event. Journal of environmental science and health Part A, Toxic/hazardous substances & environmental engineering 40: 265–287. 10.1081/ese-200045531 15717776

[54] Blahová J, Havelková M, Kruzíková K, Hilscherová K, Halouzka R, et al (2010) Assessment of contamination of the Svitava and Svratka rivers in the Czech Republic using selected biochemical markers. Environmental Toxicology and Chemistry 29: 541–549. 20821476 10.1002/etc.89

[55] Eklund B, Elfström M, Gallego I, Bengtsson BE, Breitholtz M (2010) Biological and chemical characterization of harbour sediments from the Stockholm area. Journal of Soils and Sediments 10: 127–141.

[56] Jarosova B, Blaha L, Vrana B, Randak T, Grabic R, et al (2012) Changes in concentrations of hydrophilic organic contaminants and of endocrine-disrupting potential downstream of small communities located adjacent to headwaters. Environment international 45: 22–31. 22572113 10.1016/j.envint.2012.04.001

[57] Otte JC, Andersson C, Abrahamson A, Olsman H, Keiter S, et al (2008) A bioassay approach to determine the dioxin-like activity in sediment extracts from the Danube River: Ethoxyresorufin-O-deethylase induction in gill filaments and liver of three-spined sticklebacks (Gasterosteus aculeatus L.). Environment international 34: 1176–1184. 18571727 10.1016/j.envint.2008.05.004

[58] Benedetti M, Ciaprini F, Piva F, Onorati F, Fattorini D, et al (2012) A multidisciplinary weight of evidence approach for classifying polluted sediments: Integrating sediment chemistry, bioavailability, biomarkers responses and bioassays. Environ Int 38: 17–28. 21982029 10.1016/j.envint.2011.08.003

[59] Heise S, Förstner U (2007) Risk assessment of contaminated sediments in river basins—theoretical considerations and pragmatic approach. Journal of Environmental Monitoring 9: 943–952. 17726554 10.1039/b704071g

[60] Keiter S, Braunbeck T, Heise S, Pudenz S, Manz W, et al (2009) A fuzzy logic-classification of sediments based on data from in vitro biotests. Journal of Soils and Sediments 9: 168–179.

[61] Hecker M, Hollert H (2009) Effect-directed analysis (EDA) in aquatic ecotoxicology: state of the art and future challenges. Environmental Science and Pollution Research 16: 607–613. 19705177 10.1007/s11356-009-0229-y

[62] Schüttrumpf H, Brinkmann M, Cofalla C, Frings RM, Gerbersdorf SU, et al (2011) A new approach to investigate the interactions between sediment transport and ecotoxicological processes during flood events. Environmental Sciences Europe 23: 1–5.

[63] Wetzel MA, von der Ohe PC, Manz W, Koop JHE, Wahrendorf D-S (2012) The ecological quality status of the Elbe estuary. A comparative approach on different benthic biotic indices applied to a highly modified estuary. Ecological Indicators 19: 118–129.

[64] Fischnetz (2004) On the trail of declining fish stocks. EAWAG/SAEFL, Dübendorf, Berne.

[65] Bioconsult (2009) Fischfauna des Elbeästuars - Vergleichende Darstellung von Bewertungsergebnissen nach EG-Wasserrahmenrichtlinie in den verschiedenenn Gewässertypen des Elbeästuars. Flussgebietsgemeinschaft Elbe (FGG Elbe).

